# Complete Genome Sequence of Porcine Epidemic Diarrhea Virus from an Outbreak in a Vaccinated Farm in Shandong, China

**DOI:** 10.1128/genomeA.00619-16

**Published:** 2016-07-07

**Authors:** Xiang Gao, Dongliang Li, Jingyi Zhao, Farong Xu, Xinna Ge, Xin Guo, Jun Han, Hanchun Yang, Lei Zhou

**Affiliations:** a Key Laboratory of Animal Epidemiology and Zoonosis of Ministry of Agriculture, College of Veterinary Medicine and State Key Laboratory of Agrobiotechnology, China Agricultural University, Beijing, People’s Republic of China; b Beijing Center for Animal Disease Control and Prevention, Beijing, People’s Republic of China

## Abstract

Porcine epidemic diarrhea virus, a member of the family *Coronaviridae*, is an economically important pathogen that causes severe enteritis, vomiting, dehydration, and a high mortality rate, especially among suckling piglets. Here, we report the complete genome sequence (28,036 nucleotides [nt]) of a porcine epidemic diarrhea virus (PEDV) strain isolated in a novel outbreak in Shandong, China.

## GENOME ANNOUNCEMENT

Porcine epidemic diarrhea virus (PEDV) causes severe diarrhea, vomiting, and dehydration, which lead to high mortality in suckling piglets. It was first reported in the United Kingdom in 1971 and later spread to Belgium, France, Italy, and some other European countries (1). In 1973, a transmissible gastroenteritis (TGE)-like outbreak of acute diarrhea caused by PEDV was first recorded in China (2). Since 2010, severe outbreaks with high mortality among suckling piglets have been widely reported both in Asia and North America (3–8). During May 2014 to January 2015, outbreaks of diarrhea in fattening pigs on German and Belgian farms were confirmed to be caused by two strains both genetically close to U.S. prototype strain OH851 (9, 10).

At the end of December 2014, a novel outbreak of severe diarrhea in suckling piglets occurred on a vaccinated pig farm in the Shandong Province of China. Reverse transcription-PCR (RT-PCR) directly amplifying a fragment in the M gene from feces samples was performed to demonstrate the presence of PEDV. To determine the genetic characterization and evolutionary relationship with vaccine and other field strains, the complete genome sequence of the isolated PEDV strain SD2014 was determined from extracted RNA by RT-PCR, amplifying 27 regions covering the whole genome, as described previously (4). The PCR products were gel purified and subsequently cloned into pEASY-Blunt vector (TransGen Biotech, Beijing, China), and then 3 to 5 clones of each amplicon were submitted to Sanger sequencing for determining the consensus sequence. The whole-genome sequence was assembled and analyzed using the Geneious 7 software.

The genome of isolate SD2014 is 28,036 nucleotides (nt) in length, excluding the poly(A) tail, and the genome organization is as follows: 5′ untranslated region (UTR) (nt 1 to 292), open reading frame 1a (ORF1a) (nt 293 to 12601) and ORF1b (nt 12601 to 20637) encoding a replicase protein with a ribosomal frameshift between both parts, spike (S) gene (nt 20634 to 24794), ORF3 (nt 24794 to 25468), envelope (E) gene (nt 25449 to 25679), membrane (M) gene (nt 25687 to 26367), nucleocapsid (N) gene (nt 26379 to 27704), and 3′ UTR (nt 27705 to 28036).

At the whole-genome level, strain SD2014 is most genetically close to BJ2011-1 (99.0% nucleotide similarity) isolated in China in 2011, and to the U.S. prototype strain OH851 (98.8%). However, strain SD2014 is less closely related to CV777 (96.7%) and SM98 (96.5%), from which the current vaccine strains are derived. The S gene of SD2014 only shares 93.3% and 92.5% amino acid similarity with that of CV777 and SM98, which might explain the lower protection efficiency of the vaccination.

In conclusion, the whole genome of a PEDV strain isolated from a vaccinated farm was characterized and showed highest genetic similarity with early strains isolated in 2011. This finding indicates the variant strains that are less closely related to the vaccine strain are still circulating in the fields of China.

### Nucleotide sequence accession number.

The complete genome sequence of PEDV strain SD2014 has been deposited in GenBank under the accession no. KX064280.
